# Sculpting the band gap: a computational approach

**DOI:** 10.1038/srep15522

**Published:** 2015-10-22

**Authors:** Kiran Prasai, Parthapratim Biswas, D. A. Drabold

**Affiliations:** 1 Department of Physics and Astronomy, Clippinger Laboratories Ohio University, Athens, OH 45701; 2 Department of Physics and Astronomy, The University of Southern Mississippi, Hattiesburg, MS 39406.

## Abstract

Materials with optimized band gap are needed in many specialized applications. In this work, we demonstrate that Hellmann-Feynman forces associated with the gap states can be used to find atomic coordinates that yield desired electronic density of states. Using tight-binding models, we show that this approach may be used to arrive at electronically designed models of amorphous silicon and carbon. We provide a simple recipe to include *a priori* electronic information in the formation of computer models of materials, and prove that this information may have profound structural consequences. The models are validated with plane-wave density functional calculations.

The central goal of materials science is the development of materials with novel properties. In general, this program of materials engineering has proceeded largely by experimental exploration. In this Communication, we offer a novel and direct approach to determining structures (e.g. atomic coordinates) that yield desired electronic or optical properties. The method is direct in that an initial structure is purposefully modified to drive the model toward a desired electronic density of states (for example, engineering a gap of desired magnitude, eliminating defect states in the gap, or perhaps changing the structure of band tails, a serious issue in some photo voltaic (PV) applications^1^). Such a tool is especially valuable for semiconductors, and may have value for chalcogenide phase change memory materials and device applications.

In this paper, we offer a new paradigm of computer simulation that produces models with desired optical properties and enables the inclusion of *a priori* electronic information. Naturally, there is no *a priori* guarantee that a model with preferred electronic properties should be a minimum of the total energy functional. To ensure that the model is at equilibrium, we have adopted a quench and relax procedure in which biased dynamics is employed to push the system toward a configuration with preferred electronic optical gap (by using “gap forces” described below to drive states away from the desired spectral gap into the valence or conduction bands), and slowly quenching the diffusive dynamics so that the system may explore many configurations as its dynamics is gradually arrested. The system is then relaxed with physical forces to obtain a strong energy minimum and coordinates that yield the prescribed electronic gap.

Computer models of disordered semiconductors have usually fallen short of realistically representing the material in that these models often include too many defects (e.g. miscoordinated atoms, strained bonds or misaligned bond angles). These imperfections drastically impact electronic structure of these models making them inconsistent with experimental results, usually in the form of localized electronic states in the band gap region. Recent works have shown that imposing spatial homogeneity as a modeling constraint had a dramatic effect in improving models of a-Si, solid C_60_ and other systems^2^,^3^. These workers invert the structural data (e.g. from scattering experiments) and use the reverse monte-carlo (RMC) approach to implement the constraint. We demonstrate here that “electronic homogeneity” (in particular, by eliminating localized eigenstates in the gap region) is also effective for improving the models. We offer a practical means to shepherd the eigenstates away from the gap.

We adopt tight-binding Hamiltonians and employ Hellmann-Feynman forces^4^,^5^ in a novel way to determine structures with desired optical gap. Recall that the spatially non-local part of the interatomic force, the band-structure force, has the form:


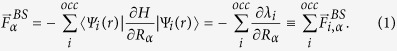


Here *i* indexes eigenvalues (or bands) and runs through all occupied states, *R*_*α*_ are the 3*N* positional degrees of freedom, *H* is the Hamiltonian, and Ψ_*i*_ is an eigenvector. If one considers individual terms in the sum in Eq. (1), the term 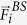 represents the contribution from the *i*^*th*^ eigenvalue (or band) to the total band-structure force. In effect, 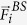 is a gradient for the *i*^*th*^ energy eigenvalue *λ*_*i*_. As such, 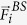 provides the direction in the 3N-dimensional configuration space of most rapid change of *λ*_*i*_. Thus, to shift *λ*_*i*_ to higher (lower) energies, we should move atoms incrementally along the direction –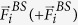. For incremental displacements *δR*_*α*_ along this gradient, the shift *δλ*_*i*_ of an eigenvalue *λ*_*i*_ can be written as 
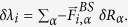
 To this end, we introduce the term *gap force* for state *i* to indicate the force (negative nuclear gradient) associated with eigenvalue *λ*_*i*_. We exploit such forces to push eigenvalues out of a spectral range that we wish to be free of states. Our modified or biased dynamics follow from a Lagrangian 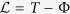, in which 
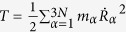
, and


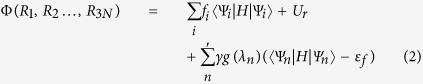


The sum in the last term in Eq. (2) is restricted to an energy range we wish to clear of states in a spectral range *λ*_*n*_ ∈ [*E*_*min*_, *E*_*max*_]. Here, *g*(*λ*_*n*_) = +1 or −1 for *λ*_*n*_ > *λ*_*HOMO*_ or *λ*_*n*_ ≤ *λ*_*HOMO*_ respectively, and *f*_*i*_ is the occupation number of *i*^*th*^ energy level, which is either 0, 1, or 2. In effect, this pushes states below the Fermi-level toward the (desired) valence edge and states above toward the conduction edge. The parameter *γ* controls the strength of the gap force, *ε*_*f*_ is the Fermi energy, and *U*_*r*_ is the repulsive ion-ion interaction. The force associated with the *α*^*th*^ degree of freedom is given by,


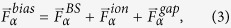


which can be used to obtain stable local minima by minimizing the total energy and forces via MD simulations and/or relaxations. In the tight-binding formulation, the forces on the right-hand side of (Eq. 3) are:


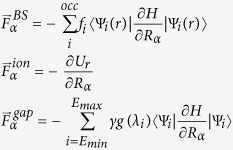


We show empirically that the method works well even for midgap states near *ε*_*F*_. We have observed that the method is also applicable in the opposite mode: to *maximize* the density of states at the Fermi level by shepherding eigenvalues toward the Fermi level. This might, for example, introduce new structural features and produce models with interesting electrical conductivity.

We demonstrate our method by modeling amorphous silicon (*a-*Si) and tetrahedral amorphous carbon (*ta-*C). These are well known to be difficult to model and to obtain a well-defined gap. To highlight the strength of our method by comparison, we also carried out identical simulations using conventional tight-binding molecular dynamics (TBMD). We created an ensemble of 25 models (with different liquid starting points) using both biased forces and conventional TBMD and then gauged these models for how well they represent the known structural and electronic properties of these materials. We observe that biased dynamics produces better models for more than four out of five cases. In the two examples below, we describe the “best” models (considering the number of four-fold atoms as the figure of merit) produced by biased dynamics (we will discuss the statistical properties of the models elsewhere). We also present the corresponding models with conventional forces. All models are then compared to the corresponding Wooten, Weire, Weiner (WWW) models^6^,^7^, which are widely recognized as best models of *a-*Si and *ta-*C. This is a proxy for comparing to experiments, since the agreement of WWW model with experiments is well established^8^,^9^,^10^. Furthermore, our results are substantiated by density functional theory (DFT) calculations^11^,^12^,^13^,^14^,^15^ in both examples.

## Results

### Disordered phases of Silicon

**Figure 1:**
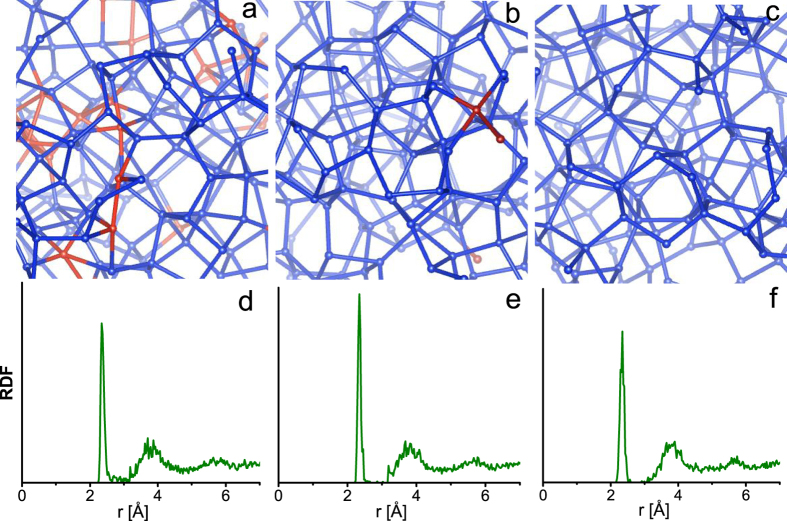
(**a**–**c**) The structural features of the TBMD, biased TBMD and WWW models of *a-*Si respectively. Mis-coordinated atoms and their bonds are visualized in red and normally coordinated atoms are in blue. Cutoff radius of 2.8 Å was used to define a bond for all three models. (**d**–**f**) Radial distribution function (RDF) of these models are plotted in the same order.

We undertake our first calculation on liquid and *a*-Si using the Goodwin-Skinner-Pettifor (GSP) Hamiltonian^16^. It is known for *a*-Si, that rapid melt-quenching from the liquid produces many gap states, remnant of the 6-fold liquid metal and an unsatisfactory model for a tetrahedral amorphous semiconductor. In this example, we impose biased dynamics favoring the creation of a gap in the range observed in WWW models^7^. After equilibrating the liquid in the conventional way, we quench with dissipative dynamics (velocity rescaling to 300 K) and biased forces. We note, for the choice of *γ* = 1 we invoke, that the average gap forces remain less than 20% of the TBMD forces on atoms: the dynamics is modified in a somewhat subtle way, but operating over many steps with diffusive dynamics, the method yields structures improved both optically (by construction) but also *structurally* (Fig. 1), a considerable bonus and proof that inclusion of electronic *a priori* information does influence structure, and in a way that improves agreement with experiments.

**Figure 2:**
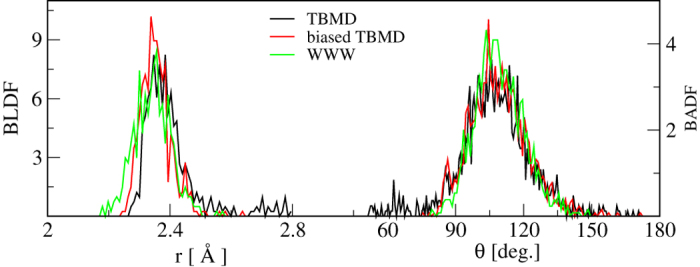
Bond-length (left) and bond-angle (right) distributions of *a*-Si from three models: biased TBMD, TBMD, and WWW^7^.

Figure 2 compares the bond-length distribution function (BLDF) and bond-angle distribution function (BADF) for three models of *a*-Si obtained from conventional TBMD, biased TBMD, and the WWW method^7^. A comparison of the radial distribution functions (RDFs) and an analysis of bond lengths shows that the biased dynamics significantly increases the short-ranged order: the biased model exhibits fewer short and long bonds as compared to conventional TBMD and WWW models. A remarkable feature of the biased-MD model is the absence of 60 degree bond angles. These angles are typically associated with frozen liquid-like configurations, which plague conventional MD simulations by producing 3-fold defects (dangling bonds). The latter are notoriously difficult to remove from MD models via conventional means and experiments show that they do not exist in significant numbers in the real material.

**Figure 3:**
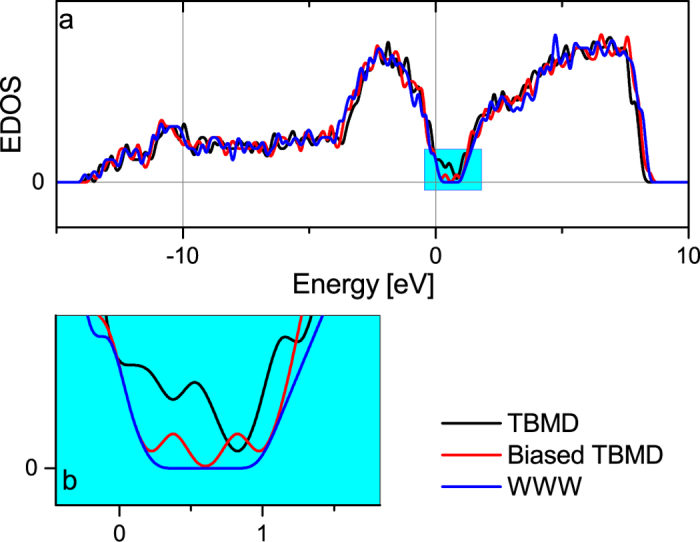
(**a**) Electronic Density of states (EDOS) for three models of *a*-Si: TBMD, biased TBMD and WWW^7^. (**b**) The density of states in the gap region is highlighted.

It is noteworthy that the inclusion of gap forces can eliminate these unphysical features completely and impart tetrahedral order in the structure. Atomic coordination is also markedly improved in biased dynamics. Atomic-coordination statistics show that 97.2% atoms in the biased-MD model are 4-fold coordinated. This result is not only superior to ~87% 4-fold coordination in conventional TBMD but also better than earlier works reported in the literature using the GSP Hamiltonian^17^,^18^,^19^. The biased-TBMD model has fewer defects around the Fermi-energy than the TBMD model (Fig. 3). Companion DFT calculations show that the energy of the best biased-TBMD model is lower than that of the conventional TBMD model. The electronic structure of these models using DFT also validates our calculation. The biased TBMD model has fewer defect states in the gap region than the TBMD model.

### Disordered phases of Carbon

As a second example we study *ta*-C. Tetrahedral amorphous carbon (*ta*-C) has some properties reminiscent of diamond while potentially holding some advantages^20^,^21^. The tight-binding model of Xu *et al.*^22^ has been used previously to model *ta*-C with limited success^23^. These calculations involved a quench from a high-density liquid (*l*-C) and volume rescaling at lower temperature. Using the same Hamiltonian, we demonstrate that a simpler melt-quench method can yield improved models. We show that our method offers a previously unavailable knob for the structural control of the model. Amorphous carbon dominated by *sp*^3^ bonding is characterized by a band gap of about 2 eV (depending on the fraction of *sp*^3^-bonded atoms) in contrast to *sp*^2^-bonded *a*-C, which has a gap of less than 0.5 eV^20^. The perfectly *sp*^3^-bonded WWW model of *ta*-C^7^, relaxed with the Xu Hamiltonian, has a gap of 4.1 eV. We used this spectral range as the gap limits to compute the gap force for biased dynamics using Eq. 3.

The unbiased TBMD with the Xu Hamiltonian prefers *sp*^2^ dominated network as observed in our calculations as well as in refs 22,24. The diamond-like *sp*^3^-bonded networks reported in^23^ appear to be an artifact of high density or high pressure on *l*-C. Our calculations produced models with up to 94% 4-fold coordination compared with 74% and 89% in ref. 23. Furthermore, our results do not involve arbitrary manipulation of density. We have conducted 25 quenching runs with different starting liquid models and all of these models produce tetrahedral networks with more than 90% 4-fold coordination.

**Figure 4:**
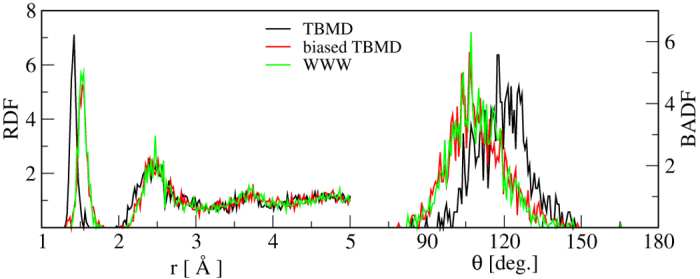
Radial (left) and bond-angle (right) distribution functions of *ta*-C from biased TBMD, TBMD, and WWW. See text for discussion of results.

**Figure 5:**
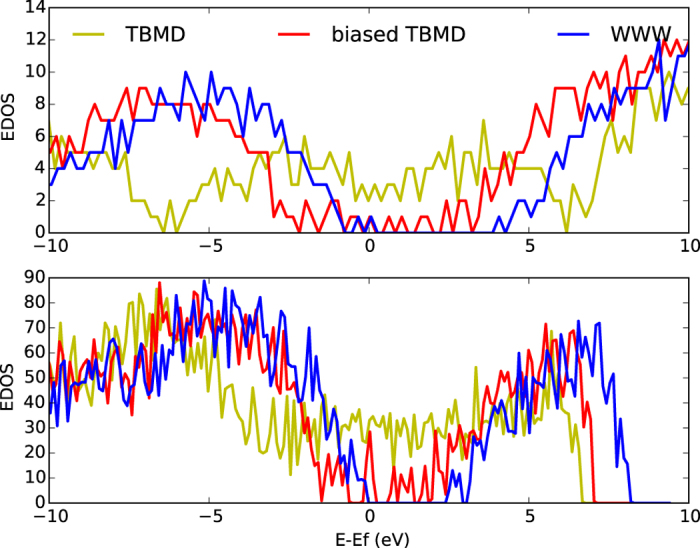
Density of electronic states (EDOS) for three models of *ta-*C: TBMD, biased TBMD and WWW^7^. The upper plot is obtained using the tight-binding model of ref. 22 and the lower one is obtained from density functional theory (DFT) calculations^11^,^12^

The structural features, including RDF, BADF and atomic coordinations from biased TBMD closely resemble with the *ta*-C WWW model. The TBMD model is dominated by *sp*^2^ bonding and registers distinct peaks in the RDF and BADF (Fig. 4). The density of electronic states of the biased-TBMD model shows that the gap opens up to 0.7 eV as compared to 0.21 eV in the TBMD model. Also, the biased-TBMD model has only 14 states in the gap region exhibited by WWW models as compared to 71 states in the TBMD model (Fig. 5). The electronic structure of these models is also confirmed by DFT calculations. The biased-TBMD model shows a few scattered states in the gap region as opposed to the ‘metal-like’ electronic structure of the TBMD model. Total energy calculations using DFT show that the biased-TBMD model is 0.31 eV/atom below the regular TBMD model. The biased model is also stable under relaxation using DFT. Such relaxation decreases the total energy by 0.07 eV/atom while preserving structural ordering of the model.

## Discussion

We demonstrate the utility of the method with two examples and suggest that the approach may be developed in promising ways. Our method offers a flexibility in modeling previously unavailable. We show in a practical way how to include electronic information in structural modeling, and we prove that imposing electronic constraints leads to relaxed models in better agreement with structural experiments, particularly for the case of *a-*Si. The method can be used to make a nearly state-free gap, or to maximize metallicity. For *ta*-C, considerable flexibility is afforded by our approach in tuning *sp*^2^/*sp*^3^ ratios. We expect that the scheme will be useful for many other complex materials not only for discovering structures with desired gaps but also for imposing electronic constraints in modeling. This scheme opens the way for new avenues of materials engineering, especially for optical properties, and we expect it to have a broad impact when implemented fully with *ab initio* methods.

### Limitations

As is the case with all methods, our approach has limitations: (1) For this first report we use standard tight-binding Hamiltonians for the simulations. Such Hamiltonians are well known to have imperfect transferability (for this reason we are currently extending the scheme to plane-wave DFT, a straightforward but tedious undertaking) and 2) even in a density-functional framework, gap estimates from Kohn-Sham eigenvalues are spurious, though usually these account reasonably well for trends. With significant computational expense, these estimates may be improved *e.g.* with GW or Hybrid Functional schemes^4^.

## Method

### Modeling *a-*Si

A 216-atom model of liquid silicon (*l-*Si) was prepared by cooling an initial random configuration from a temperature of 2500 K to the melting point ≈1780 K in several steps, which were followed by equilibration for a period of 50 ps and total-energy relaxations. We have verified that our *l-*Si model produces features similar to *l-*Si models obtained from the GSP Hamiltonian by earlier workers^17^,^18^,^25^. The *l-*Si model is equilibrated at its melting point for another 25 ps. We took 25 configurations from this run, at least 1 ps apart from each other, to begin quenching to 300 K. Each of these 25 models were quenched using biased forces as well as conventional TBMD forces. Quenching rate of ≈100 K/ps was used and, for biased dynamics *γ* = 1 was chosen. To ensure that the models are at their minimum energy with respect to the original Hamiltonian, all of these models, including those quenched to dynamic arrest using biased dynamics, were relaxed using conventional TBMD forces. Relaxation was done by slowly damping the velocity of all the atoms till the maximum force on atoms is smaller than 0.05 eV/Å. These relaxed models are reported in this paper. The energy and electronic structure of these models were also checked using DFT calculations. We used plane wave basis^11^,^12^ and PAW potentials^13^,^14^ with local density approximation (LDA)^15^. Vienna *Ab-initio* Simulation package (VASP) was used to carry out the DFT calculation.

### Modeling *ta-*C

We started with a 216-atom model of well-equilibrated model of *l-*C at density 3.5 *gm*/*cm*^3^. We equilibrated the model at 10000 K for 25 ps and sampled 25 distinct configurations of *l-*C. We quenched these models to 700 K at a rate of 500 K/ps. In the same way as first example, quenching was simulated in two ways: one with conventional TBMD forces (*γ* = 0) and the other with additional gap forces for *γ* = 1. Both quenched models were relaxed to their respective local minimum until the force on each atom is less than 0.05 eV/Å. Gap forces were switched off during post-quench relaxation for biased-TBMD models. The obtained relaxed models are reported here. Similar to the example of *a-*Si, these models were also characterized using DFT.

### Comments on statistics and technical details

1). As we have shown, our method is best employed in a “statistical mode”–unsurprisingly the final structures depend on the initial state. In some fraction (≈20%), the method does not improve the gap in case of *a-*Si. We suppose that this may be due to the very simple rule of shifting atoms along gradients toward the nearest band edge, even for eigenvalues very near *ε*_*f*_. 2) We have experimented with various *γ*, and have found no particular advantage to selecting *γ* ≠ 1 to date. 3) Preliminary studies suggest that the results presented here also accrue for larger (512-atom) models. 4) For *a*-Si, we use *a priori* knowledge of the gap from the best available models. In the general case, one can define a gap by trial and error, with the choice being determined in part by a requirement that the physical forces vanish at the end.

## Additional Information

**How to cite this article**: Prasai, K. *et al.* Sculpting the band gap: a computational approach. *Sci. Rep.* **5**, 15522; doi: 10.1038/srep15522 (2015).
